# Composted Bagasse and/or Cyanobacteria-Based Bio-Stimulants Maintain Barley Growth and Productivity under Salinity Stress

**DOI:** 10.3390/plants12091827

**Published:** 2023-04-29

**Authors:** Khadiga Alharbi, Emad M. Hafez, Alaa El-Dein Omara, Yasser Nehela

**Affiliations:** 1 Department of Biology, College of Science, Princess Nourah bint Abdulrahman University, P.O. Box 84428, Riyadh 11671, Saudi Arabia; 2 Department of Agronomy, Faculty of Agriculture, Kafrelsheikh University, Kafr El-Sheikh 33516, Egypt; 3 Department of Microbiology, Soils, Water Environment Research Institute, Agricultural Research Center, Giza 12112, Egypt; 4 Department of Agricultural Botany, Faculty of Agriculture, Tanta University, Tanta 31527, Egypt

**Keywords:** barley, cyanobacteria, composted bagasse, salinity, cereal crops, exchangeable sodium percentage (ESP), *Arthrospira platensis*, *Spirulina platensis*, *Hordum vulgare*

## Abstract

Soil and water salinity are among the most fatal environmental challenges that threaten agricultural production worldwide. This study investigated the potential impact(s) of soil amendment using composted bagasse and/or foliar application of cyanobacteria-based bio-stimulants (*Arthrospira platensis*, also known as *Spirulina platensis*) to combat the harmful effect(s) of using saline water to irrigate barley plants grown in salt-affected soils during 2020/2021 and 2021/2022. Briefly, the dual application of composted bagasse and cyanobacteria-based bio-stimulants significantly improved the soil properties, buffered the exchangeable sodium percentage (ESP), and enhanced the activity of soil enzymes (urease and dehydrogenase). Moreover, both treatments and their combination notably augmented the water relations of barley plants under salinity stress. All treatments significantly decreased stomatal conductance (*gs*) and relative water content (RWC) but increased the electrolyte leakage (EL) and balanced the contents of Na^+^ and K^+^, and their ratio (K^+^/Na^+^) of barley leaves under salinity stress compared with those irrigated with fresh water during the 2020/2021 and 2021/2022 seasons. Additionally, composted bagasse and cyanobacteria-based bio-stimulants diminished the oxidative stress in barley plants under salinity stress by improving the activity of antioxidant enzymes, including superoxide dismutase (SOD), catalase (CAT), and peroxidase (POX). Consequently, the combination of composted bagasse and cyanobacteria extract resulted in superior yield-related traits such as spike length, number of grains per spike, 1000-grain weight, grain yield, straw yield, and harvest index. Collectively, our findings suggest that the integrative application of composted bagasse and cyanobacteria is promising as a sustainable environmental strategiy that can be used to improve soil properties, plant growth, and productivity of not only barley plants but also maybe other cereal crops irrigated with saline water in salt-affected soil.

## 1. Introduction

Globally, barley (*Hordeum vulgare* L.) is grown to address the nutritional needs of human and animals. It is the fourth most important cereal crop after wheat, maize, and rice [1]. Barley is utilized as a model plant to illustrate the mechanisms of salt tolerance in monocots since it is more tolerant to abiotic stress conditions, particularly salinity stress, than other cereals [2]. It is an important crop for newly reclaimed soils, especially in the North Coastal Region of Egypt. Recently, barley has been used not only to feed humans and animals only but also in the production of malt, brew, and biodiesel [3]. The annual production in 2020/2021 was 160.53 million tons, while its production reduced to 147.05 million tons in 2021/2022 [4]. Nevertheless, irrigation with saline water in salt-affected soil and its effect on the productivity of cereal crops, such as barley, requires more investigation. 

Salinity, whether in soil or irrigation water, is one of the ineluctable environmental challenges that pose a serious danger to agricultural productivity and is regarded as a critical component that adversely impacts growth and crop yield [5]. There are more than 800 million hectares around the world affected by salinity [6]. Instead, due to extreme weather changes and ongoing agricultural practices driven by the imperative requirement for food security in light of the large population, the area grows every year [7]. Furthermore, the scarcity of sufficient water resources leads to an increase in the use of low-quality irrigation sources in agriculture, which causes soil salinization, especially given that Egypt is in an area with an aridity index of more than 60% annually, which hinders the development of sustainable agriculture [8]. Recently, the use of new agricultural approaches such as the application of bio-organic amendments has become one of the alternative ecological technologies to alleviate salt stress and its harmful effects on soil and crops [9].

The massive agricultural and industrial development accompanying the large population growth led to the accumulation of large quantities of plant wastes, which caused plenty of environmental problems, including environmental pollution caused by climate change [10]. Therefore, in pursuit of sustainable agricultural development, the recycling of organic wastes has recently become an urgent matter for scientists to reduce the harmful effects resulting from the decreased fertility of agricultural lands and the lack of water resources suitable for irrigation [11]. 

One of the significant crops suitable for Egyptian circumstances is sugarcane. Numerous tonnes of bagasse, a type of organic waste produced by sugarcane, can be used and composted [12]. It has been repeatedly and independently proved that biochar applications bring a wide range of environmental advantages [13]. Bagasse is a fibrous plant residue and one of the by-products of the sugarcane industries when sugarcane is extracted to produce juice. Composted bagasse production is low-cost, rich in nutrients, and environmentally friendly. It has a significant amount of nutrients that are essential for plant growth, which helps to improve the chemical, physical, and biological properties of the soil. It also decreases or eliminates a number of soluble and exchangeable metal fractions by enhancing metal sorption and adsorption on the surface, which helps to amend saline soils [14]. These benefits reflect positively on the growth and productivity of crops. The bagasse product is high in cellulose (45–50%), hemicellulose (27–29%), and lignin (20–23%). Bagasse contains some important micronutrients such as iron (Fe), manganese (Mn), zinc (Zn), and copper (Copper). For example, the application of silica nanoparticles from coir pith (a by-product of padding that is employed in mattress factories) enhanced the seed germination, and the shoot and root formation of *Vigna unguiculata* [15].

Due to their ability to increase plant growth and productivity, physiological and biochemical characterizations, and tolerance to abiotic challenges, bio-stimulants will soon play a crucial role in the development of sustainable agriculture [16]. Microalgae (including cyanobacterial-based bio-stimulants) are currently regarded as one of the most promising sources of new bio-stimulants, as well as an effective tool for sustainably producing a wide range of plant growth regulators and bioactive molecules such as phytohormones (auxins, cytokinins, gibberellins, and abscisic acid), vitamins, amino acids, betaines, antioxidants, polyamines, and polysaccharides. *Arthrospira platensis*, formerly known as *Spirulina*, are a filamentous blue-green alga. Cyanobacterial extracts of *A. platensis* used as foliar spraying on field crops improved nutrient absorption; enhanced anthesis and grain filling; and resulted in higher chlorophyll content and longer photosynthetically active vegetation periods, thus producing higher yield [17]. Plants treated using cyanobacterial-based bio-stimulants are more tolerant against some abiotic stress impacts such as salinity. 

Previously, we showed that plant-growth-promoting rhizobacteria (PGPR) and silica nanoparticles stimulated sugar beet resilience to salinity stress [18]. Likewise, soil amendment using biochar and glycine betaine foliar application promoted rice resilience to osmotic stress [19]. However, our knowledge about the potential roles of soil amendment using composted bagasse and the foliar application of cyanobacteria-based bio-stimulants on maintaining barley growth and productivity under salinity stress is still limited and requires further investigation. 

Based on the above reasons, our hypothesis is that the dual application of composted bagasse and cyanobacteria-based bio-stimulants might maintain barley growth and productivity under salinity stress in soil and irrigation water. Moreover, it might be environmentally and economically reasonable to use both treatments as a sustainable eco-friendly strategy to maintain soil properties, physiological attributes, biochemical characteristics, productivity, and the nutritional value of not only barley plants but also other cereal crops under salinity stress.

## 2. Results

### 2.1. Composted Bagasse and Cyanobacteria-Based Bio-Stimulants Improved the Activity of Soil Enzymes

**Figure 1:**
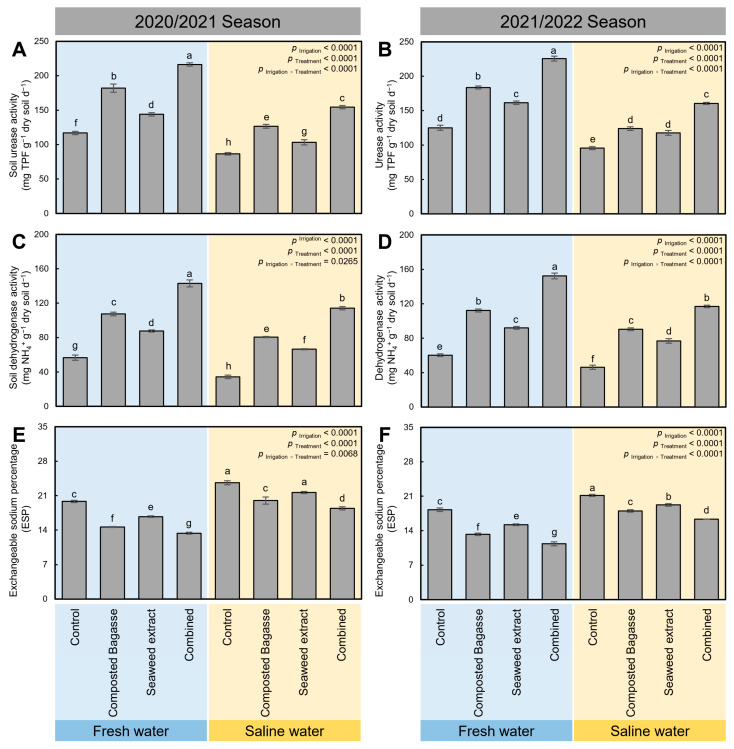
Effect of composted bagasse and cyanobacteria-based bio-stimulants on the activity of soil enzymes and exchangeable sodium percentage (ESP) under salinity stress of soil and irrigation water during the 2020/2021 and 2021/2022 seasons. (**A**,**B**) Soil urease activity (mg TPF g^−1^ dry soil d^−1^), (**C**,**D**) soil dehydrogenase activity (mg NH_4_^+^ g^−1^ dry soil d^−1^), and (**E**,**F**) exchangeable sodium percentage (ESP; %) during the 2020/2021 and 2021/2022 seasons, respectively. Bars present the means ± standard deviation (mean ± SD) of three replicates. Different letters indicate statistically significant differences between treatments according to Tukey’s HSD test (*p* _Irrigation × Treatment_ ≤ 0.05).

Although the irrigation of barley plants grown in salt-affected soil using saline water significantly reduced the activity of soil enzymes including soil urease (*p* _Irrigation_ < 0.0001; Figure 1A,B) and dehydrogenase (*p* _Irrigation_ < 0.0001; Figure 1C,D) during the 2020/2021 and 2021/2022 seasons, respectively, application of composted bagasse, a cyanobacteria-based bio-stimulant, or their combination significantly enhanced the enzymatic activity of both enzymes (*p* _Treatment_ < 0.0001). In other words, the application of composted bagasse and cyanobacteria-based bio-stimulants substantially eased the negative effect of saline water on the activity of soil enzymes.

It is worth mentioning that the dual application of composted bagasse and cyanobacteria-based bio-stimulants significantly increased the activity of urease in freshwater-irrigated soil (216.4 ± 2.41 and 225.57 ± 3.51 mg NH_4_^+^ g^−1^ dry soil d^−1^ in the 2020/2021 and 2021/2022 seasons, respectively) compared with the non-treated controls (116.95 ± 2.39 and 125.11 ± 3.60 mg NH_4_^+^ g^−1^ dry soil d^−1^ in 2020/2021 and 2021/2022 seasons, respectively). Likewise, the integrated application of both bio-stimulants enhanced the urease activity in soils irrigated with saline water (154.39 ± 2.37 and 160.50 ± 1.61 mg NH_4_^+^ g^−1^ dry soil d^−1^ in the 2020/2021 and 2021/2022 seasons, respectively) compared with the non-treated controls (86.58 ± 1.74 and 95.54 ± 2.10 mg NH_4_^+^ g^−1^ dry soil d^−1^ in the 2020/2021 and 2021/2022 seasons, respectively) (Figure 1A,B).

Similarly, the dehydrogenase activity was notably decreased after using saline water to irrigate barley plants growing in salt-affected soil during the 2020/2021 (Figure 1C) and 2021/2022 (Figure 1D) seasons. Nevertheless, the application of composted bagasse and cyanobacteria together significantly increased the activity of dehydrogenase in freshwater-irrigated soil (143.03 ± 4.07 and 152.39 ± 3.34 mg TPF g^−1^ dry soil d^−1^ in the 2020/2021 and 2021/2022 seasons, respectively) and saline water-irrigated soil (114.14 ± 1.85 and 116.99 ± 1.42 mg TPF g^−1^ dry soil d^−1^ in the 2020/2021 and 2021/2022 seasons, respectively) compared with the non-treated controls. 

### 2.2. Dual-Application of Composted Bagasse and Cyanobacteria-Based Bio-Stimulants Buffered the Exchangeable Sodium Percentage (ESP)

Although irrigation of barley plants using saline water significantly increased the exchangeable sodium percentage (ESP; *p* _Irrigation_ < 0.0001) during both seasons compared with the freshwater-irrigated plants, application of composted bagasse, cyanobacteria, or their combination significantly (*p* _Treatment_ < 0.0001) reduced the ESP during the 2020/2021 and 2021/2022 seasons (Figure 1E,F, respectively). In freshwater-irrigated soil, the ESP was reduced from 19.82 ± 0.23 and 18.25 ± 0.35% in the non-treated to 13.35 ± 0.20 and 11.31 ± 0.40% in the combination-treated soil during the 2020/2021 and 2021/2022 seasons, respectively. Likewise, in freshwater-irrigated soil, the ESP was reduced from 23.61 ± 0.42 and 21.15 ± 0.22% in the non-treated to 18.40 ± 0.32 and 16.32 ± 0.02% in the combination-treated soil during the 2020/2021 and 2021/2022 seasons, respectively (Figure 1E,F).

### 2.3. Composted Bagasse and Cyanobacteria-Based Bio-Stimulants Augmented the Water Relations of Barley Plants under Salinity Stress

**Figure 2:**
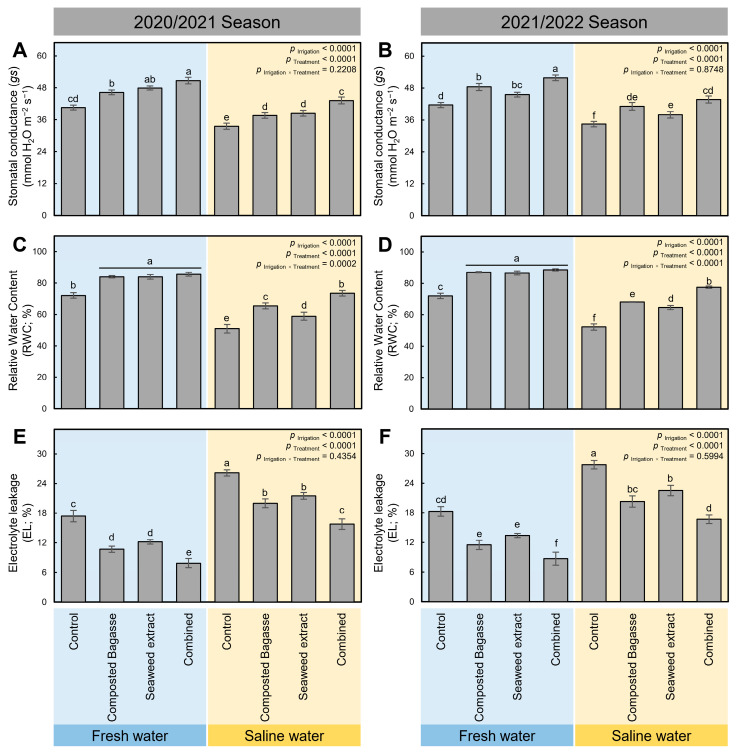
Effect of composted bagasse and cyanobacteria-based bio-stimulants on the water relations of barley plants under salinity stress in soil and irrigation water during 2020/2021 and 2021/2022 seasons. (**A**,**B**) Stomatal conductance (*gs*; mmol H_2_O m^−2^ s^−1^), (**C**,**D**) relative water content (RWC; %), and (**E**,**F**) electrolyte leakage (EL; %) during 2020/2021 and 2021/2022 seasons, respectively. Bars present the means ± standard deviation (mean ± SD) of three replicates. Different letters indicate statistically significant differences between treatments according to Tukey’s HSD test (*p* _Irrigation × Treatment_ ≤ 0.05).

In general, irrigation using saline water significantly decreased (*p* _Irrigation_ < 0.0001) stomatal conductance (*gs*; Figure 2A,B) and relative water content (RWC; Figure 2C,D) but increased the electrolyte leakage (EL; Figure 2E,F) of barley plants compared with those irrigated with fresh water during the 2020/2021 and 2021/2022 seasons, respectively. However, these devastating impacts were notably lessened after the application of composted bagasse and cyanobacteria-based bio-stimulants or their combination (*p* _Treatment_ < 0.0001). Briefly, barley plants irrigated with freshwater and treated with composted bagasse and cyanobacteria together (combined treatment) had the highest stomatal conductance (50.74 ± 1.23 and 51.86 ± 1.03 mmol H_2_O m^−2^ s^−1^ during the 2020/2021 and 2021/2022 seasons, respectively). Similarly, the combined treatment of barley plants irrigated with saline water had higher *gs* (43.2 ± 1.28 and 43.67 ± 1.31 during the 2020/2021 and 2021/2022 seasons, respectively) compared with other treatments (Figure 2A,B).

Likewise, the application of composted bagasse, cyanobacteria, or their combination significantly enhanced the RWC of barley plants irrigated with freshwater, with no significant differences between them. Nevertheless, the combined application of composted bagasse and cyanobacteria had a better influence on the RWC of barley plants irrigated with saline water than individual applications during both seasons (Figure 2C,D). On the other hand, the dual application of composted bagasse and cyanobacteria-based bio-stimulants meaningfully reduced the electrolyte leakage of barley leaves compared with that of non-treated leaves under both watering conditions (Figure 2E,F).

### 2.4. Composted Bagasse and Cyanobacteria-Based Bio-Stimulants Enhanced Chlorophyll Content (SPAD) of Barley Plants under Salinity Stress

**Figure 3:**
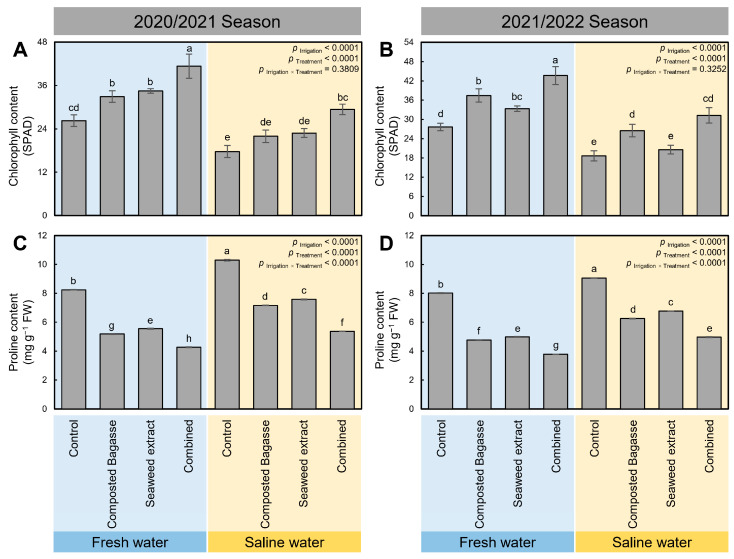
Effect of composted bagasse and cyanobacteria-based bio-stimulants on the leaf color and endogenous proline content of barley plants under salinity stress of soil and irrigation water during 2020/2021 and 2021/2022 seasons. (**A**,**B**) Chlorophyll content (SPAD) and (**C**,**D**) Proline content (mg g^−1^ FW) during the 2020/2021 and 2021/2022 seasons, respectively. Bars present the means ± standard deviation (mean ± SD) of three replicates. Different letters indicate statistically significant differences between treatments according to Tukey’s HSD test (*p* _Irrigation × Treatment_ ≤ 0.05).

Although the chlorophyll content (SPAD) of barley plants significantly decreased (*p* _Irrigation_ < 0.0001) when plants were irrigated with saline water during 2020/2021 (Figure 3A) and 2021/2022 (Figure 3B), these consequent harmful effects were significantly reduced upon treating barley plants with composted bagasse, cyanobacteria, or their combination (*p* _Treatment_ < 0.0001). For instance, the lowest chlorophyll content (SPAD) was recorded from control barley plants irrigated with saline water (17.74 ± 1.69 and 18.67 ± 1.58) in both growing seasons 2020/2021 and 2021/2022. Nonetheless, the chlorophyll content (SPAD) was dramatically increased when barley plants were treated with composted bagasse, cyanobacteria, or their combination, with the combined treatment being superior (29.39 ± 1.46 and 31.26 ± 2.42 during the 2020/2021 and 2021/2022 seasons, respectively).

### 2.5. Dual Application of Composted Bagasse and Cyanobacteria-Based Bio-Stimulants Augmented the Proline Content of Barley Plants under Salinity Stress

The endogenous proline content was significantly boosted in non-treated barley plants upon saline water irrigation (*p* _Irrigation_ < 0.0001) during 2020/2021 (Figure 3C) and 2021/2022 (Figure 3D). However, the application of composted bagasse, cyanobacteria, or their combination (*p* _Treatment_ < 0.0001) considerably decreased the proline content in barley plants irrigated with both types of irrigation water (*p* _Treatment_ < 0.0001in both seasons). Moreover, our findings showed that barley plants irrigated with saline water and treated with composted bagasse + cyanobacteria had lower proline content than individual applications of composted bagasse or cyanobacteria during 2020/2021 (Figure 3C) and 2021/2022 (Figure 3D). Similar results were observed in barley plants irrigated with freshwater upon the treatment with composted bagasse and/or cyanobacteria.

### 2.6. Composted Bagasse and Cyanobacteria-Based Bio-Stimulants Balanced the Contents of Na^+^ and K^+^, and Their Ratio (K^+^/Na^+^) in Barley Leaves under Salinity Stress

**Figure 4:**
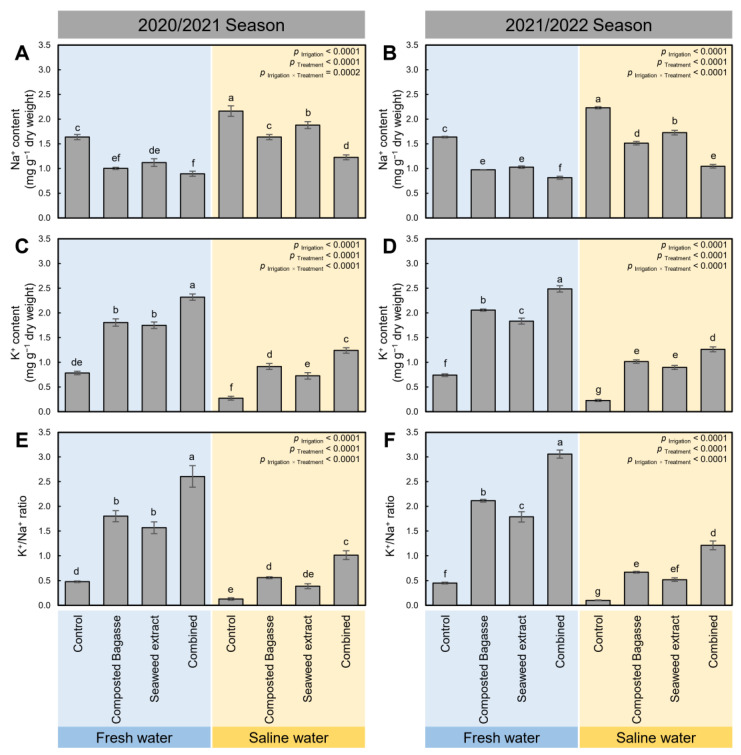
Effect of composted bagasse and cyanobacteria-based bio-stimulants on the contents of Na^+^ and K^+^, and their ratio (K^+^/Na^+^) in barley plants under salinity stress of soil and irrigation water during 2020/2021 and 2021/2022 seasons. (**A**,**B**) Na^+^ content (mg g^−1^ dry weight), (**C**,**D**) K^+^ content (mg g^−1^ dry weight), and (**E**,**F**) K^+^/Na^+^ ratio during the 2020/2021 and 2021/2022 seasons, respectively. Bars present the means ± standard deviation (mean ± SD) of three replicates. Different letters indicate statistically significant differences between treatments according to Tukey’s HSD test (*p* _Irrigation × Treatment_ ≤ 0.05).

Generally, irrigation of barley plants using saline water significantly increased the Na^+^ content (*p* _Irrigation_ < 0.0001; Figure 4A,B) but decreased the K^+^ content (*p* _Irrigation_ < 0.0001; Figure 4C,D) of barley leaves during 2020/2021 and 2021/2022, respectively. The imbalance in both Na^+^ and K^+^ contents extensively altered the K^+^/Na^+^ profile in barley plants irrigated with saline water compared with fresh-water-irrigated ones during both seasons (Figure 4E,F; *p* _Irrigation_ < 0.0001). Nevertheless, the application of composted bagasse, cyanobacteria, or their combination (*p* _Treatment_ < 0.0001) noticeably balanced the Na^+^ and K^+^, and the K^+^/Na^+^ ratio in treated barley leaves (*p* _Irrigation × Treatment_ < 0.0001 for the three parameters in both seasons). Briefly, the dual application of composted bagasse and cyanobacteria-based bio-stimulants significantly decreased the Na^+^ content but increased the K^+^ content, which resulted in a higher K^+^/Na^+^ ratio.

### 2.7. Dual Application of Composted Bagasse and Cyanobacteria-Based Bio-Stimulants Diminished the Oxidative Stress in Barley Plants under Salinity Stress

**Figure 5:**
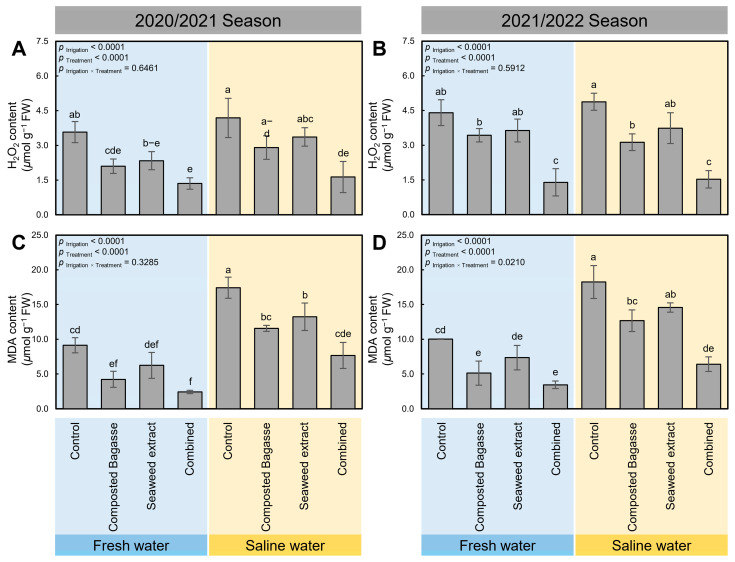
Effect of composted bagasse and cyanobacteria-based bio-stimulants on oxidative stress in barley leaves under salinity stress of soil and irrigation water during 2020/2021 and 2021/2022 seasons. (**A**,**B**) Hydrogen peroxide content (H_2_O_2_; µmol g^−1^ FW) and (**C**,**D**) malondialdehyde content (MDA; µmol g^−1^ FW) during the 2020/2021 and 2021/2022 seasons, respectively. Bars present the means ± standard deviation (mean ± SD) of three replicates. Different letters indicate statistically significant differences between treatments according to Tukey’s HSD test (*p* _Irrigation × Treatment_ ≤ 0.05).

Although irrigation of barley plants using saline water slightly increased H_2_O_2_ content (*p* _Irrigation_ < 0.0001; Figure 5A,B), it markedly boosted the lipid peroxidation (MDA; *p* _Irrigation_ < 0.0001 in both seasons; Figure 5C,D). However, the application of composted bagasse, cyanobacteria, or their combination (*p* _Treatment_ < 0.0001) notably diminished both the H_2_O_2_ and MDA contents, with a greater impact following the combined treatment during both seasons. Briefly, the dual application of composted bagasse and cyanobacteria significantly reduced the H_2_O_2_ content of freshwater-irrigated barley plants from 3.58 ± 0.46 and 4.41 ± 0.56 µmol g^−1^ FW in non-treated control plants to 1.35 ± 0.25 and 1.4 ± 0.59 µmol g^−1^ FW in combination-treated plants during the 2020/2021 and 2021/2022 seasons, respectively. A similar trend was observed in the MDA profiles of treated barley plants when irrigated with saline water in both seasons.

### 2.8. Application of Composted Bagasse and Cyanobacteria-Based Bio-Stimulants Improved the Activity of Antioxidant Enzymes of Barley Plants under Salinity Stress

**Figure 6:**
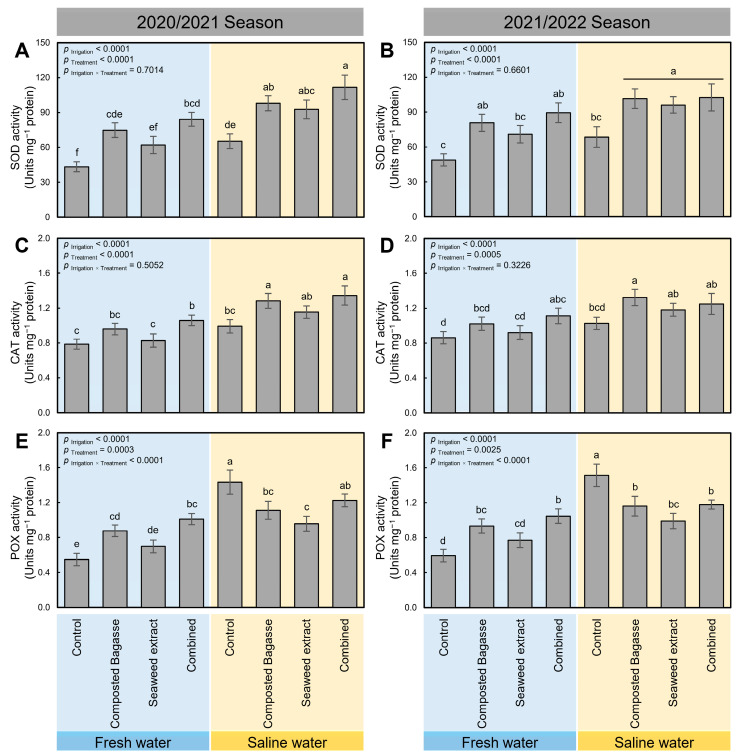
Effect of composted bagasse and cyanobacteria-based bio-stimulants on the activity of antioxidant enzymes of barley plants under salinity stress of soil and irrigation water during 2020/2021 and 2021/2022 seasons. (**A**,**B**) The activity of superoxide dismutase (SOD; unit mg^−1^ protein), (**C**,**D**) activity of catalase (CAT; unit mg^−1^ protein), and (**E**,**F**) activity of peroxidase (POX; unit mg^−1^ protein) during 2020/2021 and 2021/2022 seasons, respectively. Bars present the means ± standard deviation (mean ± SD) of three replicates. Different letters indicate statistically significant differences between treatments according to Tukey’s HSD test (*p* _Irrigation × Treatment_ ≤ 0.05).

To better understand the potential biochemical mechanisms of composted bagasse and cyanobacteria, and how they alleviate the oxidative stress in barley plants under salinity stress, the enzymatic activities of superoxide dismutase (SOD; Figure 6A,B), catalase (CAT; Figure 6C,D), and peroxidase (POX; Figure 6E,F) were evaluated during the 2020/2021 and 2021/2022 seasons. In general, using saline water to irrigate barley plants significantly increased the enzymatic activities of all three studied enzymes (*p* _Irrigation_ < 0.0001) regardless of the tested treatment. Nevertheless, the application of composted bagasse, cyanobacteria, or their combination significantly enriched the activities of all enzymes (SOD, CAT, and POX) during both seasons, with a greater effect following the combined treatment (composted bagasse + cyanobacteria).

### 2.9. Composted Bagasse and Cyanobacteria-Based Bio-Stimulants Enhanced the Yield Traits of Barley Plants under Salinity Stress

It is worth mentioning that using saline water to irrigate barley plants grown in salt-affected soils negatively altered the yield traits including spike length, grain number per spike, 1000-grain weight, grain yield, straw yield, biological yield, and harvest index (Table 1; *p* _Irrigation_ < 0.0001 for all parameters during both seasons). However, the application of composted bagasse, cyanobacteria, or their combination considerably improved all yield traits. Briefly, the dual application of composted bagasse + cyanobacteria caused the highest spike length (9.05 ± 0.03 and 9.23 ± 0.05 cm), number of grains per spike (59.28 ± 1.09 and 61.75 ± 0.74), 1000-grain weight (53.84 ± 0.39 and 56.72 ± 0.55 g), grain yield (3496.41 ± 89.82 and 3494.65 ± 35.24 kg.ha^−1^), straw yield (5277.70 ± 226.44 and 5485.68 ± 54.44 kg.ha^−1^), biological yield (8774.11 ± 145.01 and 8980.33 ± 55.61 kg.ha^−1^), and harvest index (39.87 ± 1.63 and 38.91 ± 0.38%) during the 2020/2021 and 2021/2022 seasons, respectively. Similarly, integrated composted bagasse and cyanobacteria applications to saline water-irrigated barley plants resulted in higher yield traits compared with non-treated stressed plants during the 2020/2021 and 2021/2022 seasons, respectively.

### 2.10. Application of Composted Bagasse and Cyanobacteria-Based Bio-Stimulants Enhanced the NPK Content of Barley Grains under Salinity Stress

**Figure 7:**
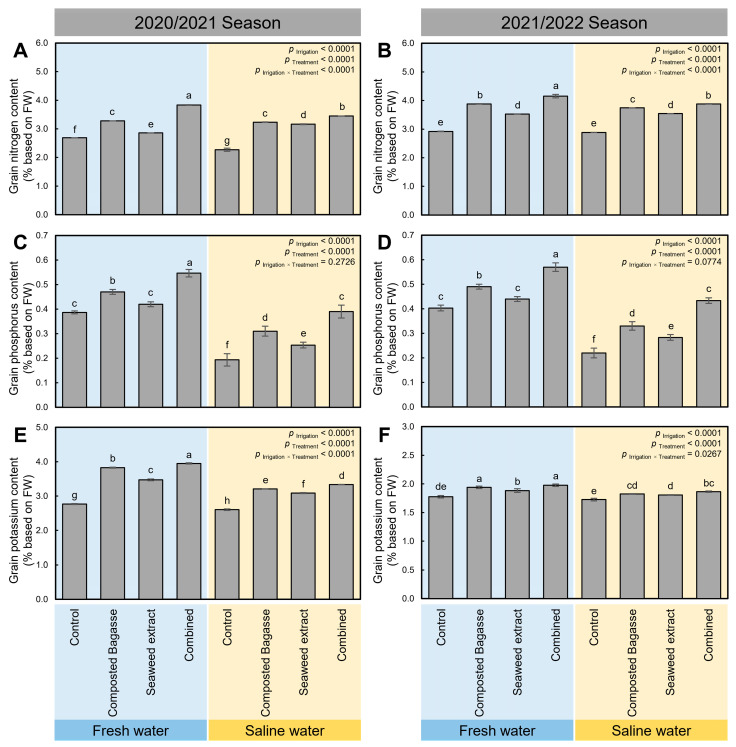
Effect of composted bagasse and cyanobacteria-based bio-stimulants on the NPK content of barley grains under salinity stress of soil and irrigation water during 2020/2021 and 2021/2022 seasons. (**A**,**B**) Grain nitrogen content (N; % based on FW), (**C**,**D**) grain phosphorus content (P; % based on FW), and (**E**,**F**) grain potassium content (K; % based on FW) during 2020/2021 and 2021/2022 seasons, respectively. Bars present the means ± standard deviation (mean ± SD) of three replicates. Different letters indicate statistically significant differences between treatments according to Tukey’s HSD test (*p* _Irrigation × Treatment_ ≤ 0.05).

Using saline water to irrigate barley plants grown in salt-affected soils significantly reduced the grain contents of nitrogen (N; Figure 7A,B), phosphorus (P; Figure 7C,D), and potassium (K; Figure 7E,F), during the 2020/2021 and 2021/2022 seasons, respectively. However, these negative effects were considerably alleviated when barley plants were treated with composted bagasse, cyanobacteria, or their combination. Both treatments significantly increased the NPK content in barley grains regardless of the type of irrigation water.

## 3. Discussion

The recent reliance on the use of low-quality water in irrigating field crops is unavoidable given the severe scarcity of available water resources, as well as the increase in soil affected by salinity due to severe climatic changes nowadays, particularly in arid and semi-arid regions, resulting in negative effects on barley plant growth and productivity. Therefore, the current study assessed the coupled application of composted bagasse and cyanobacterial-based bio-stimulants (*Arthrospira platensis*) on soil properties, physiological attributes, biochemical characteristics, growth, productivity, and nutritional value of barley plants cultivated in salt-affected soil and irrigated using saline water. Composted bagasse application has shown superior ability in reducing the Na^+^ and Cl^-^ content in the soil solution, as well as has augmented the K^+^, Ca^2+^, and Mg^2+^ contents, resulting in a reduction in exchangeable sodium percentage (ESP) [20]. The composted bagasse application enhanced the soil physical and chemical properties, as well as the soil fertility, primarily because its initial analysis includes actinomycetes, yeast, fermenting fungi, and a culture of photosynthetic and lactic acid bacteria [21]. 

The composted bagasse application improved the soil properties due to the efficient organic matter content and various elements required for plant growth as a result of increased nutrient uptake from the soil solution, which could be associated with microbial activity, resulting in improved substrate mineralization [22]. It was found that composted sugarcane bagasse is deemed a waste product; nevertheless, the current results present that it contains enough K^+^, Ca^2+^, Mg^2+^, and CEC, which positively affect the soil hydophysico-chemical characteristics, leading to enhanced soil water holding capacity and cation exchange capacity [23]. The application of composted sugarcane bagasse increased the amount of polysaccharides in the molasses as a result of the quick breakdown of organic acids, which resulted in a drop in soil pH, supporting the structure of the microbial community, and increased urease and dehydrogenase activity under salinized soil and irrigation with saline water [24]. The enhancing function of these organisms improves crop growth and productivity by supporting the plant’s ability to trap sunlight, to produce enzymes and hormones, and to accelerate the breakdown of lignin in soil [25]. 

Because a cyanobacteria extract was used in our experiment as foliar spraying to enhance physiological and biochemical processes, as well as to control oxidative stress, it had a less significant influence on soil properties than composted bagasse. However, the combined application of composted bagasse and foliar cyanobacteria extracts before planting enhanced the soil characteristics more than a solo treatment [26]. The foliar cyanobacteria extract application includes an efficient content of nitrogen and sulfur that could stimulate the photosynthesis process and, therefore, improve root growth in the soil profile, resulting in an improvement in soil properties as an indirect benefit of cyanobacteria extracts to enhance the characteristics of salt-affected soil in Egypt [27]. However, the foliar cyanobacteria extract application includes an efficient content of phosphorus and potassium and is poor in nitrogen and sulfur. Subsequently, the integration between soil amended using composted bagasse and foliar applied using a cyanobacteria extract could be used to integrate the nutritional status of salt-affected soils [28]. 

It was observed that the Phyto-availability of the necessary elements and physicochemical characteristics increased with increasing soil urease and dehydrogenase enzyme activity due to the soil amended with composted bagasse in salt-affected soil and irrigation with saline water [29]. The synthesis of phytohormones such auxins, cytokinins, and gibberellins was increased as a result of the increased root exudates in the rhizosphere, whereas the production of ethylene, ABA, and phenols was inhibited [30]. The potassium ions concentration in the leaves and the SPAD readings on account of the Na ions are favorably correlated with all the aforementioned advantages [31]. The foliar cyanobacteria extract application increased auxin and cytokinin synthesis, resulting in augmented cell division and photosynthetic capacity in terms of SPAD values [32]. 

The coupled application of composted bagasse and a cyanobacteria extract could improve the cell metabolic rate and could delay plant senescence by keeping chloroplasts from senescing due to augmented relative water contents, chlorophyll contents (SPADs), stomatal conductance, and antioxidant enzymes (SOD, CAT, and POX) and due to alleviated oxidative stress by scavenged ROS (H_2_O_2_ and MDA) and decreased electrolyte leakage and proline content, which improved plant health under these harsh conditions [33]. Barley treated with foliar spraying of a cyanobacteria extract could have sustained superior photosynthetic efficiency, which can promote sufficient metabolites to reproductive sinks for higher yield attributes and productivity [34]. The foliar cyanobacteria extract application was found to enhance the barley grain nutritional value because of the bio-regulatory impacts on enzyme activity and the translocation process from leaves to a sink. However, coupled applications gave higher increased grain nutritional values than single applications [35].

It was found that there were increased antioxidant enzymes in barley plants treated with the cyanobacteria extract. However, a coupled application increased the antioxidant enzymes more than a single application [36]. It was proved that increased SOD, CAT, and POX activities transformed hydrogen peroxide into water and oxygen, which are non-toxic compounds, leading to the protection of cell membranes and macromolecules from oxidative stress [37]. A significant decrease in yield-related traits and productivity was also found under irrigation with saline water in salt-affected soil due to a decline in assimilation, as well as a reduction in the transfer of metabolites from leaves to grains, resulting in seed sterility and a low nutritional value in grains which harmfully influenced the ovary maturity [38]. The coupled application augmented the K^+^ content, improved photosynthesis, and increased antioxidant activity, which are positively reflected in the transfer of metabolites from leaves to grains, resulting in seed fertility and nutrient uptake under irrigation with saline water in salt-affected soil [39].

The application of bagasse can increase the activity of soil enzymes such as urease and dehydrogenase, which improves soil ESP and facilitates the uptake of water and nutrients from the soil to plant tissues, which are positively reflected in the growth control of sodium ingress plants under salt stress. Additionally, the application of bagasse significantly increased the potassium influx into leaf tissues, reducing the presence of sodium ions due to the antagonism phenomenon, which resulted in increased rupture stress in leaves as a result of cell membrane stabilization and decreased electrolyte leakage. As a result, the increase in chlorophyll pigments, relative water content, and stomata conductance improved due to the application of bio-stimulants, which increases plant growth under salt-stress conditions. Furthermore, the cyanobacterial application can induce plant growth regulators within tissues such as indole-3-acetic acid (IAA) and gibberellic acid (GA3), which are instrumental in improving chlorophyll pigments’ biosynthetic, physiological, and biochemical properties due to the maintained cell membrane stability under salt pressure, which accelerated plant growth and development. The combined application of bagasse and cyanobacteria resulted in further improved organization in leaf tissues and stomatal aperture stimulated by the higher K^+^ uptake, positively affecting transpiration and increasing photosynthetic capacity, thus leading to an increase in the K^+^/Na^+^ ratio and mitigating the negative effect of salt stress. Collectively, these findings suggest a synergetic effect of the dual application of composted bagasse and cyanobacteria-based bio-stimulants. However, further studies are required to better understand how both treatments synergize with each other. 

Sugarcane bagasse biochar was proposed previously as a cost-effective source of organic matter to modulate metal and salinity stresses in soil co-contaminated with Cd and Pb under salinity stress by lowering the availability and eco-toxicity of metals following the addition of biochar [40]. Likewise, the combination of soil bagasse ash and foliar thiourea application significantly enhanced the soil properties after harvest, growth, productivity, and grain quality traits of wheat plants under different climatic conditions [41]. Additionally, foliar and soil application of bagasse compost and salicylic acid notably improved the agronomical and physiological attributes of wheat under salinity stress by reducing the toxic effects of salinity on wheat plants [42]. It is worth mentioning that biochar showed promising financial results on phosphorus recovery from sludge water and can be used to recycle phosphorus [43]. 

Our findings showed that the dual application of composted bagasse and cyanobacteria-based bio-stimulants diminished the oxidative stress in barley plants under salinity stress. This might be due to the improvement in the activity of antioxidant enzymes including superoxide dismutase (SOD), catalase (CAT), and peroxidase (POX). A techno-economic analysis of wood biochar revealed that, generally, unlike other biological wastes, it does not demand an external energy source to initiate the pyrolysis process, but it decays over time, losing many of its physical properties but boosting the soil microbial communities within the rhizosphere, which enhances soil fertility and the plant resilience to biotic and abiotic stress [44]. However, further investigations are required to better understand the physiological and molecular mechanisms behind the experimental observations, and how it is related to the metabolic profile and hormonal balance of barley plants under salinity stress and its relations with the microbial communities around barley’s roots.

## 4. Materials and Methods

### 4.1. Experimental Layout and Growth Conditions 

Two field trials were carried out to investigate the effects of composted sugarcane bagasse (CSB) and a cyanobacteria extract (CE) on the soil properties and on barley (*Hordum vulgare* L., cv. Giza 132) growth and productivity when irrigated with saline water compared with fresh water in salt-affected soil grown on 28 November in the 2020/2021 season and on 29 November in the 2021/2022 season at Elamaar village in the region of Sidi Salem (31° 07 N, 30° 57 E), Kafr El-sheik Governorate, Egypt. The experiments were conducted using four different treatments (untreated plots “control”, composted sugarcane bagasse (CSB), cyanobacteria extract (CE), and combined CSB and CE) and two types of irrigation water (fresh water and saline water). These treatments were distributed in a split-block design with three replicates, whereas two types of irrigation water were designated in the horizontal (main) plots, which were well separated to avoid infiltration when irrigation was applied, while four different treatments were assigned in the vertical (sub-main) plots. The barley seeds were obtained by the Barley Research Department, Sakha, Kafr El-Sheikh, Egypt, with an application rate of 120 kg grains ha^−1^. During the seedbed preparation pre-planting, 125 kg ha^−1^ of calcium superphosphate (15.5% P_2_O_5_) was added; in addition, 120 kg ha^−1^ of potassium sulfate (48% K_2_O) was broadcasted before the second irrigation. Furthermore, 288 kg ha^−1^ of urea (46% N) was broadcasted into two doses before the first and second irrigation. Monthly data on the maximum temperature, minimum temperature, rainfall level, and relative humidity were provided from the nearest automated weather station to the experimental farm during the 2020/2021 and 2021/2022 growing seasons (Table S1). Soil samples were taken with a soil auger at a depth of 0–30 cm and analyzed physically and chemically before cultivation (Table S2). The soil type of both locations was clayey texture and classified as salt-affected soils. The characteristics of the irrigation water (Table S3) have been described by the Soil Improvement and Conservation Department, Agricultural Research Center, Giza, Egypt.

#### 4.1.1. Composted Sugarcane Bagasse (CSB) Characterization

The sugarcane organic by-product was obtained from the sugarcane factory in Upper Egypt. It was oven-dried (70 °C) and ground to pass through a 2 mm sieve. Composted sugarcane bagasse was added to the soil before sowing at a rate of 10 tons ha^−1^, as previously recommended by Seleiman and Kheir [41]. Sugarcane bagasse was plowed into a depth of 3–5 cm in the soil. Chemical and physical characterization of the composted bagasse was analyzed according to Page et al. [45], as follows: N, 0.4%; P, 0.2%; K, 0.5%; Fe, 2.45 g kg^−1^; Mn, 124.25 mg kg^−1^; Cu, 53.48 mg kg^−1^; Zn, 41.25 mg kg^−1^; Al_2_O_3_, 2.98%; moisture content, 51.25%; dry density, 1.35 Mg m^−3^; Fe_2_O_3_, 2.22%; CaO, 12.25%; SiO_2_, 58.14%; MgO, 1.5%; specific gravity, 2.65; liquid limit, 39.55%; EC (1:10) extract, 1.75 dS m^−1^; pH (1:10) (suspension), 8.6; OM, 18.3%; and ignition loss, 2.36%.

#### 4.1.2. Cyanobacteria Extract Characterization

The fresh cyanobacteria extract (*Spirulina platensis*) was obtained from the Microbiology Department, Soils, Water and Environment Institute, Sakha Agricultural Research Station, Kafr EL-Sheikh Governorate, Egypt. It was reported previously that *Spirulina* extract at a rate of 2 g.L^−1^ was the most effective concentration to boost the growth and yield of treated plants [46]. Therefore, we used this rate (2 g of dry powder of *S. platensis* per liter) throughout this study. Briefly, two grams of dry powder from the cyanobacteria (*S. platensis*) was homogenized in 1 L of cold (4 °C) deionized water, mixed for 15 min, filtered using Whatman No. 40 filter paper from Sigma-Aldrich (Merck KGaA, Darmstadt, Germany), prepared as previously described by Dayel and El Sherif [46], and kept at −4 °C until further use. The *S. platensis* extract was foliarly applied to barley plants at the recommended dose mentioned above at 30 and 50 days.

The *Spirulina* extract has eighteen amino acids (%), i.e., alanine, 2.62; arginine, 1.96; aspartic, 3.4; cysteine, 1.71; glutamic, 3.82; glycine, 1.82; histidine, 0.45; isoleucine, 1.59; leucine, 2.55; lysine, 1.35; methionine, 1.05; phenylalanine, 1.77; proline, 1.17; serine, 1.22; tryptophan, 1.70; threonine, 2.66; tyrosine, 1.14; and valine, 2.09. The chemical composition is as follows: oligosaccharide (3%), alginic acid (5%), phytin (0.003%), menthol (5%), natural growth regulators such as cytokines (0.001%), indol acetic acid (0.0002%), pepsin (0.02%), and minerals (potassium oxide, 18%; phosphorus oxide, 5%; N, 5%; Ca, 1.5%; Zn, 0.3%; Fe, 2%; and Mn 0.1%). 

### 4.2. Measurements

#### 4.2.1. Soil Analysis

Before the beginning and the end of the trail, soil samples were collected with an auger to be air-dried, gently crushed, and sieved (2 mm) for chemical and physical analysis of the air-dried soil samples. The exchangeable sodium percentage (ESP) was calculated using the method of Seilsepour et al. [47]: ESP = 1.95 + 1.03 × SAR (R^2^ = 0.92), while SAR (sodium adsorption ratio) was calculated using the method of Richards [48], as presented in Equation (1):

(1) $$SAR=\left[Na^{+}\right]/\sqrt{\frac{\left(\left[C{a^{2}}^{+}\right]+\left[M{g^{2}}^{+}\right]\right)}{2}}$$

where Na^+^, Ca^2+^, and Mg^2+^ are stated in meq L^−1^.

#### 4.2.2. Analysis of Soil Dehydrogenase and Urease Enzymes Activity

Urease activity (mg TPF g^−1^ dry soil d^−1^) was appraised at seventy-five days after sowing based on urea as a substrate using the spectrophotometric technique at 670 nm, as provided by Kandeler and Gerber [49]. In total, 10 g of humid soil was incubated with 2 mL of methylbenzene, 11 ml of 11% urea, and 25 ml of citrate buffer (pH 6.7) for a day at 37 °C. In total, 2 mL of the purified soil solution, 2 ml of C_6_H_5_NaO, and 2 ml of NaCIO were applied and reduced to 60 ml, and absorbance was assessed at 565 nm using a spectrophotometer (RIGOL Technologies-USA, Portland, OR, USA). The dehydrogenase activity was appraised by the technique of Mersi [50]. Dehydrogenase activity was calculated with triphenyl tetrazolium chloride (TTC) as a substrate, while the triphenyl tetrazolium chloride solution (0.5–0.6 g/100 ml) was miscellaneous, with 6 g of humid soil and incubated for 24 h at 30 °C. After incubation, 50 ml of acetone was applied, and absorbance was assessed at 546 nm with a spectrophotometer. The activity of dehydrogenase was expressed as mg NH_4+_ g^−1^ dry soil d^−1^**.**

### 4.3. Physiological Characteristics 

#### 4.3.1. Leaf Relative Water Content (LRWC)

LRWC was assessed at 75 days after sowing by cutting the top-most fully expanded leaf according to the method of Weatherley [51]; the leaves were instantly weighed to obtain the fresh weight (FW). The leaves were reserved in falcon pipes (15 ml). One milliliter of distilled water was applied to each pipe and reserved in the refrigerator at 5 °C for a day. The leaves were floated on the water until a constant weight was attained in the test pipes to calculate the turgid weight (TW). The turgid leaves were oven-dried for a day at 80 °C in a ventilated oven and were weighted to obtain the dried leaves (DW). The relative water content (RLWC) was computed using Equation (2):

(2) $$RWC,\%=\frac{\left(FW-DW\right)}{\left(TW-DW\right)}\times 100$$

#### 4.3.2. Analysis of Na^+^ and K^+^ ions in Leaves 

At seventy-five days after planting, the top-most fully expanded leaf was cut according to the method Temmingho and Houba [52] to be oven-dried and digested with 8 mL of digestion mixture HNO_3_:HClO_4_ (3:1 *v*/*v*) to assess the Na^+^ and K^+^ ions and the K^+^/Na^+^ ratio as the mg g^−1^ dry weight using an ion chromatograph with a conductivity detector (Shimadzu, Japan).

#### 4.3.3. Stomatal Conductance (Sc)

At 75 days after sowing, an AP4 porometer (Headquarters address, Delta-T Devices Ltd.,130 Low Road, Burwell, Cambridge, CB25 0EJ, UK)) was used on fine days, from 9:00 to 12:00 am, on the top-most fully expanded leaves. Sc was measured from the top leaf, and the front (ra) and backside (rb) of the center of the leaf. Total leaf conductance (rl) is 1/rl = 1/ra + 1/rb and expressed as mmol H_2_O m^−2^ s^−1^.

### 4.4. Biochemical Estimation 

#### 4.4.1. Hydrogen Peroxide

Approximately 1 g of the top-most fully expanded leaves at 75 days after sowing were selected according to the method of Velikova et al. [53] to determine the H_2_O_2_ content colorimetrically, which were extracted with liquid N_2_ and trichloroacetic acid (TCA: 0.1%) centrifuged at 6000× *g* for 15 min. The concentration of the yellow supernatant was calculated at 426 nm using a spectrophotometer. 

#### 4.4.2. Malondialdehyde (MDA) 

The thiobarbituric acid test (TBA) was used to assess the MDA content using the method of Du and Bramlage [54]. At 75 days after sowing, 500 mg from the top-most fully expanded leaves was homogenized and ground in liquid N2 and a hydro-acetone buffer (4:1 *v*/*v*). So, a 20% trichloroacetic acid (TCA) solution and 0.01% butyl hydroxyl toluene (BHT) were applied, and the samples were incubated at 95 °C. After incubation, the homogenized samples were exposed to centrifugation at 10,000× *g* for 10 min. The absorbance was measured spectrophotometrically at 532 and 600 nm.

#### 4.4.3. Leaf color (SPAD)

At seventy-five days after planting, the chlorophyll values (SPAD-502, Minolta Sensing Ltd., Konica Minolta Sensing, Inc. (Headquarters: Sakai, Osaka, Japan) were used to measure the chlorophyll content (SPAD) from the top-most fully expanded leaves, as explained by Ling et al. [55]. 

#### 4.4.4. Electrolyte Leakage (EL)

The electrolyte leakage was assessed as explained by Bajji et al. [56]. Firstly, at 75 days after sowing, the electrical conductivity (EC_a_) of the ten leaf disks from fully expanded uppermost leaves was collected and washed with distilled water. Secondly, the leaf disks were put in test tubes and incubated at 55 °C for 25 min, and the electrical conductivity (EC_1_) was determined. Finally, the test tubes were boiled at 100 °C for 10 min, and the electrical conductivity (EC_2_) was measured. EL was computed using Equation (3):

(3) $$EL(\%)=\frac{\left(C1\right)}{\left(C2\right)}\times 100$$

#### 4.4.5. Proline Content

Proline is an essential osmolyte and osmo-protective compound. Leaf proline content (mg 100 g^−1^ FW) was measured using the method of Bates [57], who developed a colorimetric method to determine proline content. In total, 0.5 g of the top-most fully expanded leaves was ground with sulfuric acid (3%) and spun at 12,000 g for five minutes. The solution was quantified using a ninhydrin reagent. The obtained supernatant was then homogenized with toluene, and an absorbance of 500 was measured using a spectrophotometer at 520 nm.

### 4.5. Enzymatic Antioxidant Assays

Catalase mostly catalyzes H_2_O_2_ into H_2_O and O_2_. The measurement of CAT activity (EC:1.11.1.6) was obtained following the method of Aebi and Lester [58]. The analyzed mixture contains a plant extract, a 100 mM K-phosphate buffer (pH 7.0), and 75 mM H_2_O_2_. Therefore, the absorbance was computed at 240 nm using a UV–visible spectrophotometer. The reaction was executed by applying H_2_O_2_, and the absorbance was examined at 240 nm for 60 s. The activity of the CAT enzyme was expressed as a unit mg^−1^ protein, while the activity of SOD (EC:1.15.1.1) was calculated using the 50% NBT reduction assay at 560 nm, as explained by Beauchamp and Fridovich [59]. Superoxide dismutase (SOD) is a metalloprotein. In addition, the peroxidase (POX) (EC:1.11.1.7) activity was calculated based on o-phenylenediamine as a chromogenic marker when H_2_O_2_ and the enzyme extract were present at 417 nm, as assessed by Vetter et al. [60]. The measurements were computed as unit mg^−1^ protein.

### 4.6. Yield Traits

At harvest time, ten plants were obtained per plot to randomly measure yield attributes such as spike length (cm), number of grains per spike, and 1000-grain weight (g). Furthermore, 6 m^2^ from the inner was attained manually per plot to assess the grain and straw yield when the humidity content of the grain was 14%. Grain yields were separated with a harvester machine, oven-dried at 70 °C for a day, and weighed to determine the grain yield. The straw yield was attained by deducting the weight of grain yield from the weight of biological yield. The biological yield was estimated using the yield of the straw and grains. The harvest index (HI; %) was estimated using Equation (4):

(4) $$HI,\%=\frac{Grain\;yield\left(t\;{ha}^{-1}\right)}{Biological\;yield\left(t\;{ha}^{-1}\right)}\times 100$$

### 4.7. Nutrient Uptake

At harvest time, 50 g of grains was air-dried and subjected to 80 °C in an air-oven for two days and crushed to estimate the grain N, P, and K contents. The N, P, and K contents were determined with a micro Kjeldahl method following the technique of A.O.A.C. [61] and using a spectrophotometer and a flame photometer following the technique of Sparks et al. [62].

### 4.8. Statistical Analysis

The analysis of variance (ANOVA) was used to test the significant differences among irrigation waters (*p*_Irrigation_), treatments (*p*_Treatment_), and their interaction (*p*_Irrigation × Treatment_). Tukey’s honestly significant difference (HSD) test was used for the post hoc analysis (*p*_Irrigation × Treatment_ ≤ 0.05). An ANOVA and Tukey’s test were carried out using JMP Data analysis software, version 15 [63].

## 5. Conclusions

Collectively, our findings confirmed our hypothesis that the integrated soil amendment using composted bagasse and foliar application of cyanobacteria-based bio-stimulants noticeably improved the growth and productivity of barley plants grown in salt-affected soil and irrigated using saline water. Both treatments might be environmentally and economically reasonably used as a sustainable eco-friendly strategy to maintain soil properties, physiological attributes, biochemical characteristics, productivity, and the nutritional value of not only barley plants but also other cereal crops in salt-affected regions worldwide. Nevertheless, prospects are needed to assess the effects of different rates of these treatments on soil characteristics and productivity along with appraising these rates economically. Moreover, a comprehensive techno-economic assessment of the commercial-scale production of both treatments should be carried out to keep their costs at minimal levels.

## Figures and Tables

**Table 1:** Effect of composted bagasse and cyanobacteria-based bio-stimulants on the yield traits of barley plants under salinity stress of soil and irrigation water during 2020/2021 and 2021/2022 seasons.

Irrigation	Treatment	Spike Length (cm)	Grain Number per Spike	1000-Grain Weight (g)	Grain Yield (kg.ha^−1^)	Straw Yield (kg.ha^−1^)	Biological Yield (kg.ha^−1^)	Harvest Index (%)
2020/2021 season								
Freshwater	Control	6.99 ± 0.02 d	47.57 ± 0.46 d	43.29 ± 0.38 d	2518.56 ± 39.43 de	5072.90 ± 88.81 ab	7591.46 ± 84.74 d	33.18 ± 0.60 d
	Composted Bagasse	8.51 ± 0.12 b	55.34 ± 0.41 b	49.48 ± 0.56 b	3198.61 ± 70.28 b	5269.45 ± 101.61 a	8468.07 ± 35.00 b	37.78 ± 0.96 ab
	Seaweed extract	8.27 ± 0.03 b	51.92 ± 0.48 c	47.74 ± 0.53 c	3035.27 ± 67.42 bc	5276.66 ± 81.07 a	8311.93 ± 124.08 bc	36.52 ± 0.50 bc
	Combined	9.05 ± 0.03 a	59.28 ± 1.09 a	53.84 ± 0.39 a	3496.41 ± 89.82 a	5277.70 ± 226.44 a	8774.11 ± 145.01 a	39.87 ± 1.63 a
Saline water	Control	5.91 ± 0.07 f	38.59 ± 1.62 f	37.38 ± 0.56 f	1990.15 ± 45.37 f	4184.11 ± 77.75 c	6174.26 ± 114.17 f	32.23 ± 0.36 d
	Composted Bagasse	6.94 ± 0.09 d	46.83 ± 0.59 de	43.55 ± 0.26 d	2648.83 ± 71.42 d	4975.96 ± 155.04 ab	7624.79 ± 114.45 d	34.75 ± 1.23 cd
	Seaweed extract	6.44 ± 0.13 e	44.22 ± 1.42 e	41.10 ± 0.42 e	2347.41 ± 36.17 e	4766.26 ± 22.70 b	7113.67 ± 44.31 e	33.00 ± 0.35 d
	Combined	7.69 ± 0.16 c	50.67 ± 0.69 c	46.71 ± 0.48 c	2907.84 ± 52.21 c	5210.86 ± 136.47 a	8118.69 ± 117.87 c	35.82 ± 0.88 bc
*p* _Irrigation_		<0.0001	<0.0001	<0.0001	<0.0001	<0.0001	<0.0001	<0.0001
*p* _Treatment_		<0.0001	<0.0001	<0.0001	<0.0001	<0.0001	<0.0001	<0.0001
*p* _Irrigation × Treatment_		<0.0001	0.06996	0.0492	0.1580	0.0003	<0.0001	0.0488
2021/2022 season								
Freshwater	Control	7.10 ± 0.05 e	48.73 ± 0.43 d	44.52 ± 0.24 e	2659.84 ± 59.80 d	5107.94 ± 114.13 b	7767.79 ± 75.61 c	34.25 ± 0.95 cd
	Composted Bagasse	8.70 ± 0.06 b	56.65 ± 0.51 b	50.75 ± 0.30 b	3230.08 ± 48.64 b	5317.78 ± 133.16 ab	8547.86 ± 85.94 b	37.79 ± 0.94 ab
	Seaweed extract	8.35 ± 0.15 c	53.30 ± 0.60 c	48.76 ± 0.11 c	3040.89 ± 80.38 c	5367.91 ± 133.39 ab	8408.79 ± 53.01 b	36.17 ± 1.19 bc
	Combined	9.23 ± 0.05 a	61.75 ± 0.74 a	56.72 ± 0.55 a	3494.65 ± 35.24 a	5485.68 ± 54.44 a	8980.33 ± 55.61 a	38.91 ± 0.38 a
Saline water	Control	6.00 ± 0.08 g	40.76 ± 0.56 f	39.23 ± 0.44 g	2048.98 ± 76.77 f	4251.06 ± 67.17 d	6300.04 ± 141.73 f	32.52 ± 0.51 d
	Composted Bagasse	6.95 ± 0.08 ef	47.22 ± 0.70 de	44.42 ± 0.11 e	2642.81 ± 56.97 d	4611.65 ± 87.74 c	7254.47 ± 74.77 d	34.48 ± 0.94 cd
	Seaweed extract	6.70 ± 0.14 f	45.58 ± 0.72 e	42.40 ± 0.20 f	2418.67 ± 49.70 e	4596.44 ± 99.63 c	7015.11 ± 55.78 e	36.43 ± 0.83 bc
	Combined	7.91 ± 0.08 d	52.01 ± 0.82 c	47.71 ± 0.34 d	2883.84 ± 68.03 c	4780.25 ± 14.76 c	7664.10 ± 55.37 c	37.63 ± 0.62 ab
*p* _Irrigation_		<0.0001	<0.0001	<0.0001	<0.0001	<0.0001	<0.0001	0.0004
*p* _Treatment_		<0.0001	<0.0001	<0.0001	<0.0001	<0.0001	<0.0001	<0.0001
*p* _Irrigation × Treatment_		<0.0001	0.0327	<0.0001	0.9663	0.4947	0.2569	0.0157

Values present the means ± standard deviation (mean ± SD) of three replicates. Different letters indicate statistically significant differences between treatments according to Tukey’s HSD test (*p* _Irrigation × Treatment_ ≤ 0.05).

## Data Availability

The datasets generated and/or analyzed during the current study are available from the corresponding author upon reasonable request.

## Supplementary Materials

The following supporting information can be downloaded at https://www.mdpi.com/article/10.3390/plants12091827/s1, Table S1: Meteorological data of the experimental sites during 2020/2021 and 2021/2022 growing seasons; Table S2: Physical and chemical properties of the experimental soil (0–30 cm depth) in 2020/2021 and 2021/2022; Table S3. Characterization of irrigation water during the 2020/2021 and 2021/2022 seasons.
