# Supplementary material for: Composted Bagasse and/or Cyanobacteria-Based Bio-Stimulants Maintain Barley Growth and Productivity under Salinity Stress
Source: Plants (Basel). 2023 Apr 29;12(9):1827. doi: 10.3390/plants12091827 (PMC10181477; doi:10.3390/plants12091827)
Supplement: Supplementary file 1 [file plants-12-01827-s001.zip › plants-2367895-supplementary.pdf]

Table S1. Meteorological data of the experimental sites during 2020/2021 and 2021/2022 growing seasons.

Year Month	2020/2021				2021/2022			
	Temperature (°C)		Rainfall	Relative	Temperature (°C)		Rainfall	Relative
	max	min	(mm)	humidity (%)	max	min	(mm)	humidity (%)
December	25.8	17.4	0.68	36.3	24.4	17.5	0.74	35.6
January	24.9	15.9	1.48	34.4	23.5	15.8	0.99	33.9
February	23.4	14.7	3.68	33.6	22.6	13.9	3.51	37.8
March	22.8	11.6	6.58	44.5	21.7	10.6	6.68	46.5
April	24.4	11.9	0.42	47.5	23.8	10.3	0.47	47.2

Table S2. Physical and chemical properties of the experimental soil (0–30 cm depth) in 2020/2021 and 2021/2022.

Character	2020/21	2021/22
pH (1:2.5 soil:water suspension)	8.11 ± 0.01 †	8.12± 0.01
Electrical conductivity (EC, dS m^{-1}) ¥	5.14 ± 0.01	5.25 ± 0.01
Soil organic matter (g kg^{-1})	11.22 ± 0.01	11.35 ± 0.02
ESP # (%)	22.01 ± 0.14	21.08± 0.11
Soluble cations (meq L^{-1}) ¥		
Ca^{++}	7.25 ± 0.44	8.01 ± 0.26
Mg^{++}	5.14 ± 1.22	6.09 ± 1.19
Na^{+}	26.65± 2.01	24.25 ± 3.01
K^{+}	0.67 ± 0.08	0.55 ± 0.09
Soluble anions (meq L^{-1}) ¥		
CO_3^{--}	nd ‡	nd
HCO_3^{-}	4.33 ± 0.23	3.98 ± 0.15
Cl^{-}	24.44 ± 1.01	22.65 ± 1.35
SO_4^{--}	15.54 ± 3.32	14.36 ± 3.55
Available macronutrients (mg kg^{-1})		
N	9.78± 0.24	9.12 ± 0.18
P	8.98 ± 1.36	8.87 ± 1.45
K	325 ± 20.03	335 ± 24.28

† Standard deviation; ‡ not detected; ¥ measured in soil paste extract; # exchangeable sodium percentage.

Table S3. Characterization of irrigation water during the 2020/2021 and 2021/2022 seasons.

Character	Fresh Water		Saline Water *	
	2020/21	2021/22	2020/21	2021/22
pH	7.65 ± 0.44	7.48 ± 0.52	8.21 ± 0.09	8.02 ± 0.08
EC (dS m^{-1})	0.68 ± 0.11	0.77 ± 0.01	3.97 ± 0.04	3.98 ± 0.05
SAR	1.48 ± 0.11	1.48 ± 0.06	7.69 ± 0.47	7.88 ± 0.35
Na^+ (mq L^{-1})	1.65 ± 0.12	1.74 ± 0.04	15.36 ± 1.21	16.03 ± 1.19
Cl^- (mq L^{-1})	3.52 ± 0.01	3.63 ± 0.05	12.22 ± 0.58	11.89 ± 0.41
SO_4^- (mq L^{-1})	0.19 ± 0.02	0.18 ± 0.02	7.57 ± 0.17	8.10 ± 0.05
NH_4^+ (mq L^{-1})	1.49 ± 0.03	1.32 ± 0.04	2.35 ± 0.02	2.21 ± 0.03
COD (mq L^{-1})	12.39 ± 0.77	11.91 ± 0.96	nd ¥	nd
BOD (mq L^{-1})	5.28 ± 0.38	5.33 ± 0.37	nd	nd
SS (mq L^{-1})	184 ± 12.59	180 ± 12.4	16 ± 1.2	15 ± 1.2
DS (mq L^{-1})	378 ± 44	387 ± 45	2854 ± 148	2789 ± 145

COD: chemical oxygen demand; BOD: biological oxygen demand; SS: suspended solids; DS: dissolved solids. * Well water at a depth of 20 m; ¥ not detected.
